# Prevalence and Clinical Characteristics of Aquagenic Pruritus among Medical and Pharmacy Students in Lomé (Togo)

**DOI:** 10.1155/2020/8420123

**Published:** 2020-05-21

**Authors:** Julienne Noude Teclessou, Koussake Kombate, Abla Sefako Akakpo, Abas Mouhari-Toure, Julie Zoua, Panawe Kassang, Bayaki Saka, Palokinam Pitche

**Affiliations:** ^1^Department of Dermatology—Venerology CHU Campus, Lomé. Faculty of Health Sciences, Lomé University, Lomé, Togo; ^2^Department of Dermatology—Venerology CHU Sylvanus Olympio, Lomé. Faculty of Health Sciences, Lomé University, Lomé, Togo; ^3^Department of Dermatology—Venerology CHU Kara. Faculty of Health Sciences, Kara University, Kara, Togo

## Abstract

**Objective:**

The aim of this study was to determine the prevalence and clinical characteristics of aquagenic pruritus (AP) in medical students in Lomé (Togo).

**Methods:**

This was a prospective and descriptive study conducted among medical students in Lomé from June 1^st^ to August 30^th^, 2019. The data collection questionnaire was anonymous composed of sociodemographic variables, bathing habits, and history of allergy responding to the concept of aquagenic pruritus and its characteristics.

**Results:**

In our study, 129/591 medical students had AP, giving a prevalence of AP to 21.8%. The average age of students with AP was 23.9 years, and the M/F sex ratio was 1.5. AP was not present after each bath in 100% of the medical students who suffered from it and lasted an average of 9.09 minutes. It was characteristically pruritic (60.5%) or tingling (38.0%) and localized (45.0%) or generalized (55.0%) in respondents with history of AP. There was a significant association between the presence of AP and a personal history of allergic rhinitis (*p* < 0.01) and the presence of AP and a family AP (*p* < 0.01). Twenty-six (20.2%) respondents with AP feared taking a bath. Bathing with warm or lukewarm water (29.5%) or applying menthol ointment (27.1%) were the main precautions taken to reduce AP.

**Conclusion:**

Aquagenic pruritus is a common condition in medical students in Togo. It occurs mainly in males and can be familial.

## 1. Introduction

Aquagenic pruritus (AP) is a dermatologic affection characterized by unpleasant itching associated to contact with water, without cutaneous lesion [1]. AP can be idiopathic or associated with various systemic diseases such as Hodgkin lymphoma, myelodysplastic syndrome, essential thrombocythemia, and polycythemia vera (Vasquez disease) [2]. In some families, AP appears to be hereditary [3]. AP manifests as pruritus and tickling or stinging (burning) sensation all over the body or at localized parts as chest or proximal parts of the limbs [3, 4].

Although AP is especially described in childhood and adolescence, it can occur in all age groups and in both sexes, with a male to female sex ratio of up to 1.7 in favor of males [3]. The exact prevalence of AP is not well known. A prevalence of 4.5% was reported in Israel [5] and in Nigeria 21% to 23.5% [6, 7].

To our knowledge, there are no previous studies carried out on AP in Togo. The aim of our study was to evaluate the prevalence of aquagenic pruritus and its clinical characteristics in a young population composed of medical students in Lomé (Togo).

## 2. Method

We carried out a prospective and descriptive study among students of the Faculty of Health Sciences (FHS) in the University of Lomé (UL) for 3 months: from 1^st^ June to 30^th^ August 2019. The University of Lomé is the biggest public university in Togo comprising fifteen faculties, one of which is the FHS. The FHS includes medicine, pharmacy, and a postdoctoral school.

Lomé is the capital of Togo and the largest urban town of the country. Pipeborne water is distributed by the Togolese Water Company, the sole water distributor, to households who can afford it. However, this distribution is only available downtown. So, in rural areas, households generally use water from personal boreholes or rain water which has been collected and stored in reservoirs for many weeks. Sometimes, people with personal boreholes sell water in bowls.

Concerning cosmetics, most of the soaps commonly used are industrially manufactured and imported, and sold in different places (market, pharmacy, and beauty institutes). There exist locally manufactured soaps which are generally made of soda or ash from burnt wood. Body creams are mostly imported, but sometimes, shea butter is used as a body cream.

A previously planned and tested, autoadministered, and anonymous questionnaire was sent to medical students by e-mail. The e-mail addresses were obtained from the medical and pharmacy students associations of the FHS.

We considered as AP when any students presented itching or tingling after water contact in the bath.

The study variables were as follows:

1. Sociodemographic data
2. Personal or family history of atopy
3. Bath habits: the water sources used, the water's temperature, the type of soap, and the body cream or lotion used after the bath
4. Other contacts with water (swimming pool)
5. The clinical characteristics of aquagenic pruritus: onset, the duration, and the localization on the body
6. The practice or therapy used to calm the pruritus

Comparison of variables such as personal atopy and AP history, personal and family history of atopy, and bath habits, i.e., the water source, water's temperature used for the bath, the type of soap, and the cream used on the body after the bath were analyzed with respect to the presence or not of AP.

Data were analyzed using Epi Info 7 software. For continuous variables, mean and standard deviation was calculated. Pearson's chi-squared test was used in bivariate analysis. The significance threshold was set at 5%.

## 3. Results

A total of 591 students answered and returned the questionnaires. The mean age was 23.0 ± 14.0 with a range of 17 to 33 years old, and the male to female sex ratio was 0.54. The students lived in various places in the town, and only 6/591 (1.0%) lived on the university campus.

129/591 (21.8%) students presented AP; their mean age was 23.9 ± 21.03 years old with a range from 17 to 32 years old. 78/129 (60.5%) students were male with a male to female sex ratio of 1.5.

Duration of AP was 7.3 ± 5.2 years (1 month to 20 years). 30% (39/129) of students presented with AP appearing 3 to 18 minutes after the bath, with a mean of 5.72 minutes (Table 1). The mean duration of AP was 9.09 minutes ± 8.6 with a range of 3 to 60 minutes. All the participants (100%) presenting AP said there was not pruritus after each bath. Nevertheless, AP occurred often in 21 (16.3%) of our participants (Table 1).

78/129 (60.5%) of students presented pruritus sensation after the bath, and 49/129 students (38%) presented tickling. Generalized pruritus occurred in 71/129 students (55%), while localized pruritus occurs in 58/129 students (45%). The most frequent location of AP on the body was on the limbs (36% and 27%) (Table 1). Itching occurred also in 71/129 (55%) participants when they were exposed to rain. Bathing with lukewarm or warm water (38% and 29.5%, respectively) or mentholated ointment application (27.1%) were the main precautions to reduce the occurrence of AP. Antihistaminics (anti-H1) reduced the pruritus in 14/129 (10.9%) (Table 1).

A comparison between AP and non-AP students showed association with a personal history of allergic rhinitis and the occurrence of AP (*p* < 0.01) (Table 2).

## 4. Discussion

AP is an affection characterized by unpleasant itching associated to contact with water. AP is a frequent affection in the general population of Lomé with a prevalence of up to 21.8%. Our results are similar to those of Salami et al. in Nigeria who found a prevalence of 23.8% among students [6]. Olumide et al. also reported a prevalence of 21% in Nigeria in a hospital [7]. These results reveal the high prevalence of this affection in the general population in Africa. The exact prevalence of aquagenic pruritus should be determined by multicentric studies.

In our study, we observed a male predominance (of AP) with an M/F sex ratio of 1.5. The male predominance was also reported in Nigeria with a sex ratio of 1.7 [6].

The duration of evolution of AP was 7.3 years with a maximum of 20 years. This shows that it is a chronic affection. AP did not occur after each bath in respondents because 100% of them said that they were not itching themselves after each bath. However, the long duration of evolution of affection (until 20 years) cannot exactly precise the regularity of occurring pruritus after the bath.

AP can occur just few minutes after the bath. As to the onset of pruritus with the bath, it started 3 to 18 minutes after the bath in 30% of affected students (with a mean of 5.7 minutes) in our study. AP is defined in the literature as a pruritus which generally occurs immediately or many hours after contact with water [8]. Our result is concordant with this definition.

An AP episode lasted an average of 9.09 minutes in our participants. Salami et al. [6] in Nigeria reported a mean AP time of 5.5 minutes, while Potasman et al. [5] in Israel observed that AP occurred in 5 minutes following water contact in 76% cases and lasted for 10 to 30 minutes. Our results are different from those of Steinman et al. [1] who reported a mean AP duration time of 40.6 minutes. Although the physiopathologic mechanism is not very well known, the variation of durations of AP in different studies could be suggestive of the interference of other factors which maintain the pruritus after its onset by water contact.

A history of allergic rhinitis was the most frequent history of atopy in 24/129 (18.6%) medical students with AP. There is a significant association between the presence of AP and the personal history of allergic rhinitis (*p* < 0.01).

Allergic conjunctivitis (18.75%) followed by allergic rhinitis (12, 5%) were the main allergic histories reported by Salami et al.in Nigeria [6].

A significant association was also found between the presence of AP and a familial AP (*p* < 0.01). Family tendency was also reported [3].

The origin and temperature of water and the quality of soap used were not associated with the onset of AP in our study. Steinman et al. [1] reported that AP occurred after contact with cold, lukewarm, or warm water. However, 53/129 (41.1%) of AP-affected participants in our study said symptoms occurred after spending time in the rain.

The occurrence of AP upon contact with any source of water shows that it is ubiquitous in character.

Participants, 26/129 (20.2%), with AP in our study feared taking baths, and 34 (26.4%) reduced their bathing time. Our results are higher than those of Salami et al. in Nigeria [6] who found that 8.33% of AP-affected people did not want to take a bath. So, AP can alter the life quality of affected people.

To reduce AP, 35/129 (27.1%) applied mentholated ointment; 14/129 (10.9%) used antihistaminic drugs, and 16/129 (12.4%) wore clothes immediately after the bath. Salami et al. [6] also reported that 6.25% of AP-affected patients used antihistaminic drugs and 10.42% of them immediately wore clothes after the bath. Wearing clothes immediately after the bath could reduce the AP sensation time. On the other hand, therapeutic assays with antihistaminic drugs on AP patients were vain [8].

### 4.1. Limitations

This study presents 2 limits. Firstly, students did not consult to search cutaneous lesions associated to AP. Secondly, we did not perform blood tests (or another paraclinical exams) in order to check a systemic cause of AP.

## 5. Conclusion

Aquagenic pruritus is a frequent affection in the study population in Togo. It occurs mainly in males. AP starts immediately or few minutes following a bath and can be generalized or localized in some parts of the body. Family cases of AP have been reported, so it is therefore important to search for a genetic factor which intervenes in the affection.

## Figures and Tables

**Table 1:** Aquagenic pruritus characteristics.

Characteristics	*n*	Percentage (%)
How often (at which frequency) do you itch yourself after the bath?		
Sometimes	95	73, 6
Often	21	16, 3
Rarely	13	10, 1

When (in which moment) do you feel AP sensation?		
In the bath	1	0, 8
Just after the bath	89	69, 0
The minutes following the bath	39	30, 2

How do you qualify this sensation?		
Itching/pruritus	78	60.5
Tickling	49	38.0
Stinging/burning	2	1.5

In which part of your body occurs AP?		
All the body	71	55.0
Chest	22	17.1
Limbs	36	27.9
Hand palms/foot palms	0	0.0

Does the duration of scratching depend on duration of the bath?		
Yes	23	17.8
No	106	82.2

What other daily habits can trigger this same sensation as after bathing?		
Be/stay in rain	71	55.0
Sweating	53	41.1
Swimming	18	13.9

Do you fear bathing because of AP?		
Yes	26	20.2
No	103	79.8

Do you reduce your bath time/duration because of AP?		
Yes	34	26.4
No	95	73.6

Which habits/precautions reduce your AP?		
Bath with lukewarm/warm water	38	29.5
Ointment/cream application after bathing^*∗*^	35	27.1
Reducing the bath number per day	26	20.1
Quickly get dressed/wearing clothes	16	12.4
Bath without sponge/net	16	12.4
Taking drugs	14	10.9
Nothing	45	34.9

^*∗*^Mentholated ointment/cream.

**Table 2:** Aquagenic pruritus associated factors.

Characteristics	Aquagenic pruritus				
	Present	Absent
	*n*	*n*	OR	IC	*p*
Atopy history					
Asthma	3	25	0.41	0.12–1.40	0.14
Eczema (atopic dermatitis)	34	105	1.21	0.77–1.89	0.39
Allergic rhinitis	**24**	**105**	**2.78**	**1.57–487**	**0.0002**
Familial atopy's history	68	60	1.28	0.86–1.90	0.28
Familial aquagenic pruritus	**60**	**69**	**4.15**	**2.72–6.32**	**0.0001**
Do you swim	36	93	1.04	0.67–1.61	0.84

Bath habits					
Bath with bore or well water	69	60	0.68	0.45–1.01	0.05
Bath with TDE water	56	73	1.29	0.87–1.92	0.20
Bath with rain water	9	120	0.86	0.40–1.83	0.69
Bath with ambient temperature	96	32	1.32	0.84–2.05	0.22
Bath with lukewarm/warm water	50	79	1.19	0.79–1.78	0.38
Bath with synthetic net	117	11	0.92	0.45–1.87	0.83
Bath with traditional sponge	7	122	0.88	0.37–2.08	0.78
Bath with washcloth	12	117	1.37	0.68–2.75	0.3
Bath with antiseptic soap	17	112	0.73	0.41–1.27	0.28
Bath with glycerin soap	31	98	0.88	0.55–1.38	0.58
Bath with soda/wood ash soap	28	101	1.52	0.92–2.47	0.08
Bath with other soaps (perfumed, whitening)	53	76	0.98	0.66–1.47	0.95
Body ointment/cream application after bathing	48	80	0.93	0.61–1.39	0.73
Immediately get dressed after bathing	91	38	0.83	0.54–1.28	0.40

## Data Availability

The data relating to this manuscript are available in the documentation center of the Department of Dermatology, University of Lomé.
